# Editorial: Exploring cats: their behaviors and human-cat interactions

**DOI:** 10.3389/fvets.2023.1329398

**Published:** 2023-12-05

**Authors:** Emma K. Grigg, Dennis C. Turner, Leslie A. Lyons, Benjamin L. Hart, Lynette A. Hart

**Affiliations:** ^1^Department of Population Health and Reproduction, School of Veterinary Medicine, University of California, Davis, Davis, CA, United States; ^2^School of Animal and Comparative Biomedical Sciences, University of Arizona, Tucson, AZ, United States; ^3^Institute for applied Ethology and Animal Psychology, Horgen, Switzerland; ^4^Department of Veterinary Medicine and Surgery, College of Veterinary Medicine, University of Missouri, Columbia, MO, United States; ^5^Department of Anatomy, Physiology, and Cell Biology, School of Veterinary Medicine, University of California, Davis, Davis, CA, United States

**Keywords:** cats, welfare, human-animal interactions, free-roaming cats, human-animal bond, companion animals

## Introduction

Domestic cats are immensely popular companion animals in households around the world (1). Over 45 million US households contain at least one companion cat (2); in the European Union, the population of pet cats is estimated to be 113 million (outnumbering the estimated 92 million dogs) (1). Despite this global popularity, research into the behavior and welfare of cats living in private homes is still limited; and arguably, even less is known about the mechanisms of human-cat interactions within the home. Outside the home, cats allowed uncontrolled outdoor access alongside free-roaming cat colonies outside of human ownership (but not always without human care), still generate considerable controversy between animal advocates and conservationists concerned about cats' impact on wildlife. This Research Topic presents 12 new papers that shed light on these issues and more. The goal of this Research Topic is to improve our understanding of companion cats, with particular focus on their interactions with humans, and human attitudes toward these animals. The twelve manuscripts in this Research Topic on cat behaviors and the development of the human–cat bond cover a wide variety of themes.

## The mechanics of human-cat interactions

Turner(a) presents a mini-review of the available literature on a number of topics relevant to our understanding of human-cat interactions, such as the importance of kitten socialization, how cats communicate with their humans, and the mechanics of social interactions between cats and humans (such as the influence of who initiates contact, and of symmetry in compliance, or lack of compliance, with the partner's “wishes”). Noting the importance of ensuring the animals' wellbeing during human-cat interactions (and the scarcity of research into this issue with companion cats), Haywood et al. present Human-Cat Interaction guidelines designed to improve the comfort and welfare of companion cats during such interactions. They developed and tested the efficacy of these new guidelines with 100 shelter cats, interacting with 120 novel members of the public, and report their results here. Nagasawa et al. examine physiological (urinary oxytocin and cortisol) responses to interaction with humans, by comparing these variables in cats during positive interactions with a familiar caretaker (including physical contact, play, etc.), vs. when such interactions were removed. In another study using physiological (fecal cortisol) measures of stress, along with weight and behavior, Carlisle et al. investigate stress levels of cats adopted by families of children with autism spectrum disorder (ASD). Cats in the Carlisle et al. study were specifically selected for sociability and calmness using the validated Feline Temperament Profile, and the adopters provided with education on cat behavior; the authors discuss the importance of these factors to successful adoption into these homes.

## Understanding interactions between cats

Gajdoš Kmecová et al. review and seek to extend the existing research on play in cats, much of which has focused to date on object play [e.g., (3)], by looking at social play between cats. They suggest using a psychobiological approach to the study of play, which considers the motivational and emotional states of the cats; and present an ethogram (synthesized from the literature) and common terminology for use in future studies of cat social play. Khoddami et al. also seek to extend the existing literature on interactions between cats in multi-cat homes, by focusing specifically on two-cat households. They note that previous studies frequently lack focus on any particular group size, limiting our understanding of social dynamics in specific group sizes, despite the fact that most multi-cat households in the US and Canada consist of two cats (4, 5).

## Free-roaming cats and wildlife

Four papers in this issue focus on free-roaming cats, with two exploring the often-contentious issue of domestic cats' impact on wildlife. Tan et al. summarize the arguments for and against allowing cats outdoor access, and identify several owner- and cat-related factors associated with allowing companion cats uncontrolled access to the outdoors. Kim et al. investigate attitudes of different demographic groups toward feral cats in Seoul, South Korea, following the establishment of government-supported cat feeding stations around that city. They report distinct and sometimes complex differences between the groups in their attitudes toward cats and their preferred management approach for feral cat populations [e.g., trap-neuter-release (TNR) vs. culling]; they also discuss the possible impact of the feeding stations on these results. Turner(b) takes a critical look at the literature on cats' impacts on wildlife, in light of recent media reports of the “alarming predation of house cats on prey populations.” Turner(b) cautions that researchers should avoid bias and misinterpretation of field data, by considering what is known about predatory behavior in domestic cats and reporting estimates of total prey species population sizes. In their paper examining human-cat interactions involving free-living cats, Wandesforde-Smith et al. note the “moral pluralism” involved in the emphasis (even requirement) for humane care and protection of owned companion cats, alongside the systematic culling of large numbers of cats supported by public policy.

## Use of technology in research on cats

The final two papers discuss research into new use of technology in the study of domestic cats. Xu et al. apply and advocate for machine learning techniques (in contrast to the more traditional biomechanical experiments with living cats or cat cadavers) for improving our understanding of the feline “athletic ability.” Given recent work using heart rate variability (HRV) as an indicator of emotion in non-human animals [e.g., (6)], Grigg et al. compare HRV data collected using an affordable, commercially available cardiac monitoring system (Polar H10) against data from a traditional ambulatory electrocardiogram, to assess whether the Polar monitors could be used for this purpose in unrestrained cats.

## In summary

This Research Topic tackles a broad range of topics relevant to domestic cats. Many of the papers add particular insight into our understanding of human-cat, and cat-cat, interactions. Others report on issues important to cat welfare, such as controversies surrounding outdoor cats and wildlife. Our understanding of domestic cat behavior and human-cat interactions continues to improve, as these papers demonstrate.

## Author contributions

EG: Writing – original draft, Writing – review & editing. DT: Writing – review & editing. LL: Writing – review & editing. BH: Writing – review & editing. LH: Writing – review & editing.

## References

[1] HealthforAnimals.org. Global State of Pet Care. (2021). Available online at: https://www.healthforanimals.org/reports/pet-care-report/global-trends-in-the-pet-population/#worldwide (accessed February 13, 2023).

[2] American Pet Products Association. 2021-2022 APPA National Pet Owners Survey. Stamford, CT: APPA (2022).

[3] Hall SL Bradshaw JWS. The influence of hunger on object play by adult domestic cats. Appl Anim Behav Sci. (1998) 58:143–50. 10.1016/S0168-1591(97)00136-6

[4] Canadian Federation of Humane Societies (CFHS). Cats in Canada 2017: A Five-Year Review of Cat Overpopulation. (2017). Available online at: https://humanecanada.ca/wpcontent/uploads/2020/03/Cats_In_Canada_ENGLISH.pdf (accessed February 13, 2023).

[5] Larkin M,. Pet Population Still on the Rise, With Fewer Pets Per Household. American Veterinary Medical Association. (2021). Available online at: https://www.avma.org/javma-news/2021-12-01/pet-population-still-rise-fewer-pets-household (accessed February 13, 2023).

[6] Von Borell E Langbein J Despres G Hansen S Leterrier C Marchant-Forde J. Heart rate variability as a measure of autonomic regulation of cardiac activity for assessing stress and welfare in farm animals – a review. Physiol Behav. (2007) 92:293–316. 10.1016/j.physbeh.2007.01.007 17320122

